# Lipidome characterisation and sex-specific differences in type 1 and type 2 diabetes mellitus

**DOI:** 10.1186/s12933-024-02202-5

**Published:** 2024-03-29

**Authors:** Maria Barranco-Altirriba, Núria Alonso, Ralf J. M. Weber, Gavin R. Lloyd, Marta Hernandez, Oscar Yanes, Jordi Capellades, Andris Jankevics, Catherine Winder, Mireia Falguera, Josep Franch-Nadal, Warwick B Dunn, Alexandre Perera-Lluna, Esmeralda Castelblanco, Didac Mauricio

**Affiliations:** 1 Department of Endocrinology & Nutrition, Hospital de la Santa Creu i Sant Pau, Barcelona, Spain; 2 Departament d’Enginyeria de Sistemes, Automàtica i Informàtica Industrial, Universitat Politècnica de Catalunya, B2SLab, Barcelona, Spain; 3 Networking Biomedical Research Centre in the subject area of Bioengineering, Biomaterials and Nanomedicine, CIBER-BBN), Madrid, Spain; 4 Institut de Recerca Sant Joan de Déu, Esplugues de Llobregat, Barcelona, Spain; 5 CIBER de Diabetes y Enfermedades Metabólicas Asociadas, Instituto de Salud Carlos III, Barcelona, Spain; 6 Servicio de Endocrinología y Nutrición, Hospital Universitario e Instituto de Investigación en Ciencias de la Salud Germans Trias i Pujol, Badalona, Barcelona, Spain; 7 Departament de Medicina, Universitat Autònoma de Barcelona (UAB), Barcelona, Spain; 8 School of Biosciences, University of Birmingham, Edgbaston, Birmingham, B15 2TT UK; 9 Phenome Centre Birmingham, University of Birmingham, Edgbaston, Birmingham, B15 2TT UK; 10 Institute of Metabolism and Systems Research, University of Birmingham, Edgbaston, Birmingham, B15 2TT UK; 11 Department of Endocrinology & Nutrition, Hospital Universitari Arnau de Vilanova, Institut de Recerca Biomèdica de Lleida (IRBLleida), Lleida, Spain; 12 Department of Electronic Engineering, Universitat Rovira i Virgili, IISPV, Tarragona, Spain; 13 Institut Investigació Sanitària Pere Virgili (IISPV), Tarragona, Spain; 14 Biomolecular Mass Spectrometry and Proteomics, Bijvoet Center for Biomolecular Research, Utrecht Institute for Pharmaceutical Sciences, University of Utrecht, Utrecht, The Netherlands; 15 Netherlands Proteomics Center, Utrecht, The Netherlands; 16 Centre for Metabolomics Research, Department of Biochemistry, Cell and Systems Biology, Institute of Systems, Molecular and Integrative Biology, University of Liverpool, L69 7ZB Liverpool, UK; 17 Institut d’Investigació Biomèdica, Centre Atenció Primària Cervera, Gerència d’Atenció Primària, Universitat de Lleida, Institut Català de la Salut, Lleida, Spain; 18 DAP-Cat Group, Unitat de Suport a La Recerca Barcelona Ciutat, Institut Universitari d’Investigació en Atenció Primària Jordi Gol, Barcelona, Spain; 19 Division of Endocrinology, Metabolism and Lipid Research, Department of Internal Medicine, Washington University School of Medicine, 63110 St. Louis, MO USA; 20 Unitat de Suport a la Recerca Barcelona, Institut Universitari d’Investigació en Atenció Primària Jordi Gol i Gurina, 08007 Barcelona, Spain; 21 Institut d’Investigació Biomèdica Sant Pau (IR Sant Pau), 08041 Barcelona, Spain; 22 Faculty of Medicine, University of Vic, Vic, Spain

**Keywords:** Untargeted lipidomics, Type 1 diabetes, Type 2 diabetes, Sex-specific differences

## Abstract

**Background:**

In this study, we evaluated the lipidome alterations caused by type 1 diabetes (T1D) and type 2 diabetes (T2D), by determining lipids significantly associated with diabetes overall and in both sexes, and lipids associated with the glycaemic state.

**Methods:**

An untargeted lipidomic analysis was performed to measure the lipid profiles of 360 subjects (91 T1D, 91 T2D, 74 with prediabetes and 104 controls (CT)) without cardiovascular and/or chronic kidney disease. Ultra-high performance liquid chromatography-electrospray ionization mass spectrometry (UHPLC-ESI-MS) was conducted in two ion modes (positive and negative). We used multiple linear regression models to (1) assess the association between each lipid feature and each condition, (2) determine sex-specific differences related to diabetes, and (3) identify lipids associated with the glycaemic state by considering the prediabetes stage. The models were adjusted by sex, age, hypertension, dyslipidaemia, body mass index, glucose, smoking, systolic blood pressure, triglycerides, HDL cholesterol, LDL cholesterol, alternate Mediterranean diet score (aMED) and estimated glomerular filtration rate (eGFR); diabetes duration and glycated haemoglobin (HbA1c) were also included in the comparison between T1D and T2D.

**Results:**

A total of 54 unique lipid subspecies from 15 unique lipid classes were annotated. Lysophosphatidylcholines (LPC) and ceramides (Cer) showed opposite effects in subjects with T1D and subjects with T2D, LPCs being mainly up-regulated in T1D and down-regulated in T2D, and Cer being up-regulated in T2D and down-regulated in T1D. Also, Phosphatidylcholines were clearly down-regulated in subjects with T1D. Regarding sex-specific differences, ceramides and phosphatidylcholines exhibited important diabetes-associated differences due to sex. Concerning the glycaemic state, we found a gradual increase of a panel of 1-deoxyceramides from normoglycemia to prediabetes to T2D.

**Conclusions:**

Our findings revealed an extensive disruption of lipid metabolism in both T1D and T2D. Additionally, we found sex-specific lipidome changes associated with diabetes, and lipids associated with the glycaemic state that can be linked to previously described molecular mechanisms in diabetes.

**Supplementary Information:**

The online version contains supplementary material available at 10.1186/s12933-024-02202-5.

## Introduction

Diabetes mellitus (DM) is characterized by chronic hyperglycaemia that leads to heterogenous disturbances of metabolism [1] and its continuing rise is a major concern in society [2]. DM is one of the main risk factors for cardiovascular disease and other conditions [3]. Therefore, a better understanding of diabetes pathophysiology has become a subject of major interest in research.

Lipid disruption has been associated with many human diseases, leading to the rise in the relevance of lipidomics, an emerging field involving the study of lipids and factors that interact with lipids [4]. The association between lipids and DM has been widely recognised. Advanced lipoprotein analyses have shown a reduction in serum concentrations of triglycerides (TG), cholesterol, and apolipoprotein (Apo)B-containing lipoproteins when comparing subjects at the onset of T1D and after achieving optimal glycaemic control [5]. In addition, epidemiological studies have shown a close relationship between low-density lipoprotein cholesterol (LDL-cholesterol) and high-density lipoprotein cholesterol (HDL-cholesterol) concentrations in T2D. However, the complexity of the associations between diabetes and lipid metabolites is underestimated in these studies since lipoproteins contain a great variety of lipids that remain unanalysed, highlighting the crucial role lipidomics can play [6].

The association between lipid species and T1D still needs to be fully understood. Most of the studies have focused on biomarker discovery for T1D risk during childhood [7]. One study reported several lipid species significantly altered in subjects at the onset of T1D and after achieving glycaemic control [8]. On the other hand, the number of studies focused on lipidomic changes associated with T2D risk is higher [9–12]. Lipidome differences between normoglycemic, prediabetic and T2D subjects have also been described [13], as well as lipid species significantly associated with T2D complications, such as diabetic retinopathy [14], diabetic neuropathy [15] or diabetic nephropathy [16].

Nevertheless, to the best of our knowledge, there is a lack of studies comparing the lipidome of subjects with T1D and T2D. Moreover, some studies have shown evidence that the risk of diabetes complications differs between the sexes [17]; however, the underlying mechanisms behind these sex-specific differences are poorly understood.

In the present study, we extensively investigated the serum lipidome of T1D, T2D, and non-diabetic subjects through an untargeted lipidomics analysis using Ultra High-Performance Liquid Chromatography-Mass Spectrometry (UHPLC-MS). Our objectives were to identify lipid subspecies that differ between (1) subjects with T1D and with T2D, (2) subjects with T1D and non-diabetic subjects, (3) subjects with T2D and non-diabetic subjects, (4) to determine sex-specific differences in each of the above-mentioned comparisons, and (5) compare the lipidome between normoglycemia, prediabetes and T2D.

## Methods

### Participants

In this study, 536 participants including 156 with T1D, 159 with T2D, and 221 without diabetes and matched by sex and BMI, were selected from previous cohorts, at the University Hospitals Arnau de Vilanova (Lleida, Spain), Germans Trias i Pujol (Badalona, Spain), Clinic (Barcelona, Spain), and the Primary Care Center Mollerussa (Lleida, Spain) [18–21] (Additional File1 - Figure S1). The inclusion criteria for all groups were: aged between 20 and 85 years, the absence of established chronic kidney disease (defined as calculated glomerular filtration rate < 60 mL/min and/or urine albumin/creatinine ratio > 299 mg/g), and absence of known clinical cardiovascular events or associated revascularization procedures, including coronary heart disease, cerebrovascular disease, or peripheral vascular disease (including the diagnosis of diabetic foot disease).

Age, sex, tobacco exposure and pharmacological treatment were recorded. Diabetes duration was acquired from the medical records. Subjects were considered to have hypertension or dyslipidaemia if they were under anti-hypertensive or lipid-lowering treatment, respectively. Anthropometric data, weight, height, waist circumference, BMI, and blood pressure were obtained using standard methods. The standard biochemical analysis included glucose and glycated hemoglobin (HbA1c), lipid profile, and estimated glomerular filtration rate calculated according to the Chronic Kidney Disease Epidemiology Collaboration (CKD-EPI) equation [22]. The dietary pattern was assessed using the alternate Mediterranean Diet score (aMED), as described previously [23]. This score includes monounsaturated-to-saturated fat ratio, legumes, vegetables, nuts, fruits, nuts, cereals, fish, meat and wine. This score ranges from 0 to 9, with higher scores indicative of a higher adherence to the MedDiet.

Blood samples were collected in the fasting state and blood tests were conducted using standard laboratory methods [18]. Urine tests were performed in subjects with diabetes following standard laboratory methods. Subjects with normoglycemia and prediabetes were classified using the American Diabetes Association criteria (glycated haemoglobin (HbA1c) < 5.7% or fasting plasma glucose ≤ 100 mg/dL for normoglycemia; and HbA1c between 5.7% and < 6.5% or fasting plasma glucose between 101 mg/dL and < 126 mg/dL for prediabetes) [24].

Blood samples for the lipidomic analyses were collected in the fasting state with EDTA tubes, processed immediately after extraction, and stored at − 80 °C at the biobanks of the participant centres until determination.

From the 536 samples, 23 were discarded due to technical problems, and for the present study, the lipid profiles of 360 participants, 91 with T1D, 91 with T2D and 178 without diabetes were selected from the full cohort (Additional File 1 - Figure S1). Serum samples for all the selected participants were collected by University Hospital Arnau of Vilanova (Lleida, Spain) and the Mollerussa Primary Health care area (Lleida, Spain), as part of previously published studies [18–20]. The acquisition, processing and storage of these samples were performed in the same facility, and the geographic location of the subjects was the same, thus avoiding any variability due to sample origin that might be present in the full cohort (Additional File 1 - Figure S1).

### Sample preparation

Due to the large number of samples in the full cohort, samples were randomly assigned to one of 6 batches. To reduce the impact of technical factors, the sample order within each batch was randomized before sample preparation, and then again prior to measurement of the lipid profile by UHPLC-ESI-MS/MS. All serum samples were defrosted on ice, and each sample was aliquoted (50 µL) to create a pooled quality control (QC) representative of all samples in the study. The pooled QC was vortexed, further aliquoted (50 µL), and stored at -80 °C until the analysis of each of the 6 batches of QC samples. Lipid extraction was performed by mixing 50 µL of biological sample or QC with 150 µL isopropanol (LC-MS grade), vortexed for 20 s, and centrifuged at 22,000 g for 20 min at 4 °C. 120 µL of the supernatant was transferred to a low recovery vial and transferred to the LC sample manager at 4 °C.

### Ultra-high-performance liquid chromatography-mass spectrometry

Samples were maintained at 4 °C and analysed by applying UHPLC-MS methods using a Dionex UltiMate 3000 Rapid Separation LC system (Thermo Fisher Scientific, MA, USA) coupled with a heated electrospray Q Exactive Focus mass spectrometer (Thermo Fisher Scientific, MA, USA). Non-polar extracts were analysed on a Hypersil GOLD column (100 × 2.1 mm, 1.9 μm; Thermo Fisher Scientific, MA, USA). Mobile phase A consisted of 10 mM ammonium formate and 0.1% formic acid in 60% acetonitrile/water and mobile phase B consisted of 10 mM ammonium formate and 0.1% formic acid in 90% propan-2-ol/water. Flow rate was set for 0.40 mL/min with the following gradient: t = 0.0, 20% B; t = 0.5, 20% B, t = 8.5, 100% B; t = 9.5, 100% B; t = 11.5, 20% B; t = 14.0, 20% B, all changes were linear with curve = 5. The column temperature was set to 55 °C and the injection volume was 2µL. Data were acquired in positive and negative ionization mode separately within the mass range of 150–2000 *m/z* at resolution 70,000 (FWHM at *m/z* 200). Ion source parameters were: sheath gas = 50 arbitrary units, Aux gas = 13 arbitrary units, sweep gas 3 arbitrary units, spray voltage 3.5 kV (positive ion mode) and 3.1 kV (negative ion mode), Capillary temp = 263 °C, and Aux gas heater = 425 °C. Data dependent MS2 in ‘Discover mode’ was applied for the MS/MS spectral acquisition with the following settings: resolution at 17,500 (FWHM at *m/z* 200), isolation width 3.0 *m/z*, stepped normalised collision energy at 20, 50 and 80%. Spectra were acquired at three mass ranges 200–400 *m/z*, 400–700 *m/z* and 700–1500 *m/z* on the pooled QC samples. Thermo ExactiveTune (2.8 SP1 build 2806) software was used to control the instrument in both cases, with data acquired in profile mode. Quality control samples were acquired in both profile and dependent scan mode at the start of the run (i.e., 7 QCs MS1 only, 3 QCs with MS2) and then every seventh injection with two QC samples at the end of the analytical batch. Preparation blank samples were analysed between QCs 5 and 6 and at the end of the analytical batch.

### Mass spectrometry raw data processing

Raw data acquired in each analytical batch were converted from the instrument-specific format to a mzML file format using the open access ProteoWizard (version 3.0.11417) msconvert tool [25]. Deconvolution was performed by the R package XCMS (version 1.46.0, running in the Galaxy workflow environment) [26]. Isotopologue Parameter Optimization (IPO - version 1.0.0) [27] was used to optimise the XCMS peak picking parameters. A data matrix of metabolite features (m/z-retention time pairs) versus samples was constructed with peak areas provided.

### Assessment of data quality and peak matrix filtering

The first five QCs for each batch were used to equilibrate the analytical system and therefore subsequently removed before the data was processed and analysed. Data matrices were corrected for run-order drift in intensity for each lipid feature separately using the Quality Control-Robust Spline Correction (QC-RSC) algorithm [28] in the R environment using the pmp package [29]. Principal Component Analysis (PCA) was used to identify and remove (PCs 1 and 2, Hotelling T^2^*p* < 0.05) suspected outlier (QC) samples within each batch to ensure robust correction. Blank samples at the start and end of a run were used to remove features from non-biological origins. Any feature with an average QC intensity less than 20 times the average intensity of the blanks was removed. Any sample with > 50% missing values was excluded from further analysis. Metabolite features with RSD > 30% and present in less than 90% of the QC samples were deleted from the dataset. Features with a < 50% detection rate over all samples were also removed. All data preparation steps were undertaken in R using the structToolbox package [30, 31].

### Statistical analysis

After removing the observations with missing values in the variables included in the models (Additional File 1 - Figure S1), the clinical data of participants was summarised as mean (standard deviation) for continuous variables and as frequency (percentage) for categorical data, using the compareGroups R package [32].

Analysis of the UHPLC-ESI-MS/MS data was conducted in the R environment [33]. Prior to the statistical analysis, Probabilistic Quotient Normalization (PQN) [34], using the mean of the QC samples as a reference, was applied. Data were log-transformed to reduce skewness.

Multiple linear regression models were used to assess the association between each metabolite and T1D, T2D, and non-diabetic controls (CT). Three different comparisons were performed: T1D against T2D, T1D against CT, and T2D against CT. The models were adjusted by sex, age, hypertension, dyslipidaemia, body mass index, glucose, smoking, systolic blood pressure, TG, HDL cholesterol, LDL cholesterol, alternate Mediterranean diet score and estimated glomerular filtration rate (eGFR); diabetes duration and glycated haemoglobin (HbA1c) were also included in the T1D and T2D comparison.

Regarding the sex-specific differences related to diabetes, the same linear models were used, but an interaction term between diabetes and sex (basal level: men) was added. Using this configuration, the p-value and regressor (β_Men_) associated with the diabetes variable were assigned to men. The effect of diabetes in women (β_Women_) was computed by summing the regressor of the diabetes variable and the interaction between diabetes and sex. The standard error of the effect was computed, the t-value was obtained by dividing β_Women_ by its standard error, and the p-value associated with diabetes in women was computed using this t-value.

Concerning the lipid alterations associated with the glycaemic state, we considered the following categories: normoglycemia, prediabetes and T2D. The models were adjusted using the confounding factors mentioned above, and a numeric variable defining the glycaemic state: 0, normoglycemia; 1, prediabetes; 2, T2D.

In all analyses, False Discovery Rate (FDR) correction was performed, and a corrected p-value of < 0.05 was considered significant. For each comparison, we present significant p-values that correspond to lipids when not considering the interaction effect between diabetes and sex (all subjects), p-values for men, p-values for women, and p-values for the glycaemic state.

A description of each analysis is shown in Additional File 1 (Table S1). LipidSearch was used to annotate lipid species. Annotations with grades A or B were mapped to XCMS-detected features based on an absolute ppm error less than 5 and an absolute retention time tolerance of less than 5 s.

## Results

### Clinical and biological parameters

The baseline characteristics for each group according to diabetes status are shown in Table 1. Subjects with T1D had longer diabetes duration, and higher HDL-cholesterol in comparison with subjects with T2D. On the other hand, subjects with T2D were older, had a higher BMI, higher HbA1c, and higher frequency of hypertension and dyslipidaemia. In Additional File 1 (Table S2), the baseline characteristics for each comparison are shown.

**Table 1:** Baseline characteristics of subjects with type 1, type 2 diabetes, prediabetes and normoglycemia

	Control	Prediabetes	T1D	T2D	P-value
	*N* = 100	*N* = 69	*N* = 59	*N* = 86	
**Sex (men)**	60 (60.0%)	34 (49.3%)	27 (45.8%)	46 (53.5%)	0.308
**Age (years)**	53.1 (12.3)	56.7 (11.9)	50.7 (9.9)	58.0 (10.0)	< 0.001
**Hypertension (yes)**	17 (17.0%)	19 (27.5%)	22 (37.3%)	48 (55.8%)	< 0.001
**Dyslipidemia (yes)**	26 (26.0%)	30 (43.5%)	30 (50.8%)	49 (57.0%)	< 0.001
**BMI (kg/m** ^**2**^ **)**	25.7 (3.6)	27.8 (4.2)	25.5 (3.6)	31.7 (5.6)	< 0.001
**Waist circumference (cm)**	94.5 (11.7)	99.3 (11.4)	89.1 (12.2)	106 (12.4)	< 0.001
**DM duration (years)**	.	.	24.6 (10.4)	10.6 (7.7)	< 0.001
**HbA1c (%)**	5.26 (0.28)	5.73 (0.31)	7.58 (0.94)	8.16 (1.54)	< 0.001
**Glucose (mg/dL)**	88.0 (7.1)	95.3 (9.5)	154.0 (79.4)	169.0 (61.2)	< 0.001
**Smoking (former/current)**	41 (41.0%)	42 (60.9%)	31 (52.5%)	52 (60.5%)	0.024
**sBP (mmHg)**	122 (15)	129 (14)	130 (19)	139 (18)	< 0.001
**dBP (mmHg)**	77 (10)	80 (9)	71 (10)	77 (11)	< 0.001
**TG (mg/dL)**	105.0 (50.6)	124.0 (58.3)	68.9 (38.1)	127.0 (65.8)	< 0.001
**HDL-cholesterol (mg/dL)**	58.0 (15.2)	56.3 (11.3)	65.5 (16.8)	49.0 (12.7)	< 0.001
**LDL-cholesterol (mg/dL)**	128.0 (29.2)	127.0 (31.1)	102.0 (20.2)	103.0 (33.8)	< 0.001
**Insulin (yes)**	0 (0.0%)	0 (0.0%)	59 (100%)	31 (36.0%)	< 0.001
**Metformin (yes)**	0 (0.0%)	0 (0.0%)	0 (0.0%)	68 (79.1%)	< 0.001
**aMED**	3.38 (1.81)	3.07 (1.59)	4.36 (1.61)	4.19 (1.51)	< 0.001
**eGFR (mL/min/1.73 m** ^**2**^ **)**	87.2 (14.9)	83.6 (18.3)	91.7 (14.3)	89.8 (19.2)	0.033

*Data are mean (SD) for continuous variables and number (%) for categorical variables. For continuous variables, the p-values are obtained using a student’s t-test and for categorical variables, a chi-squared test. BMI, body mass index; DM, diabetes mellitus; HbA1c, glycated haemoglobin; sBP, systolic blood pressure; dBP, diastolic blood pressure; TG, triglycerides; HDL, high-density lipoprotein; LDL, low-density lipoprotein; aMED, alternate Mediterranean diet score; eGFR, estimated glomerular filtration rate.*

### Lipid markers of diabetes mellitus

Table 2 shows the overall number of lipidomic features that were significantly different in the comparisons (corrected p-value lower than 0.05), and of those, the number that were annotated using LipidSearch. When comparing T1D and T2D subjects, 30 lipid species (positive and negative ion modes combined) obtained a corrected p-value lower than 0.05 both in males and females (all subjects), 16 lipids were found in females and 17 in males. In the comparison between T1D and CT, 35 lipids were statistically significant in all subjects, 11 lipids in women only, and 16 in men only. Finally, when comparing T2D vs. CT, 15 lipids were significantly different in all subjects, 10 in women only, and 10 in men only. Overall, 54 unique lipid species from 15 classes were determined as significant features across all comparisons.

UpSet plots depicting the number of unique and shared (i.e., intersections) significant lipidomic features in the different comparisons in positive and negative acquisition modes are shown in Additional File 1 (Figures S2 and S3), respectively.

**Table 2:** Summary of the number of significant lipidomic features in each comparison

Comparison	All subjects	Women	Men
T1D vs. T2D	638 (30)	374 (16)	213 (17)
T1D vs. CT	1053 (34)	527 (11)	538 (16)
T2D vs. CT	450 (15)	292 (10)	282 (10)

Number of significant features in each comparison (number of annotated significant features that fulfil the annotation criteria)

**Fig. 1:**
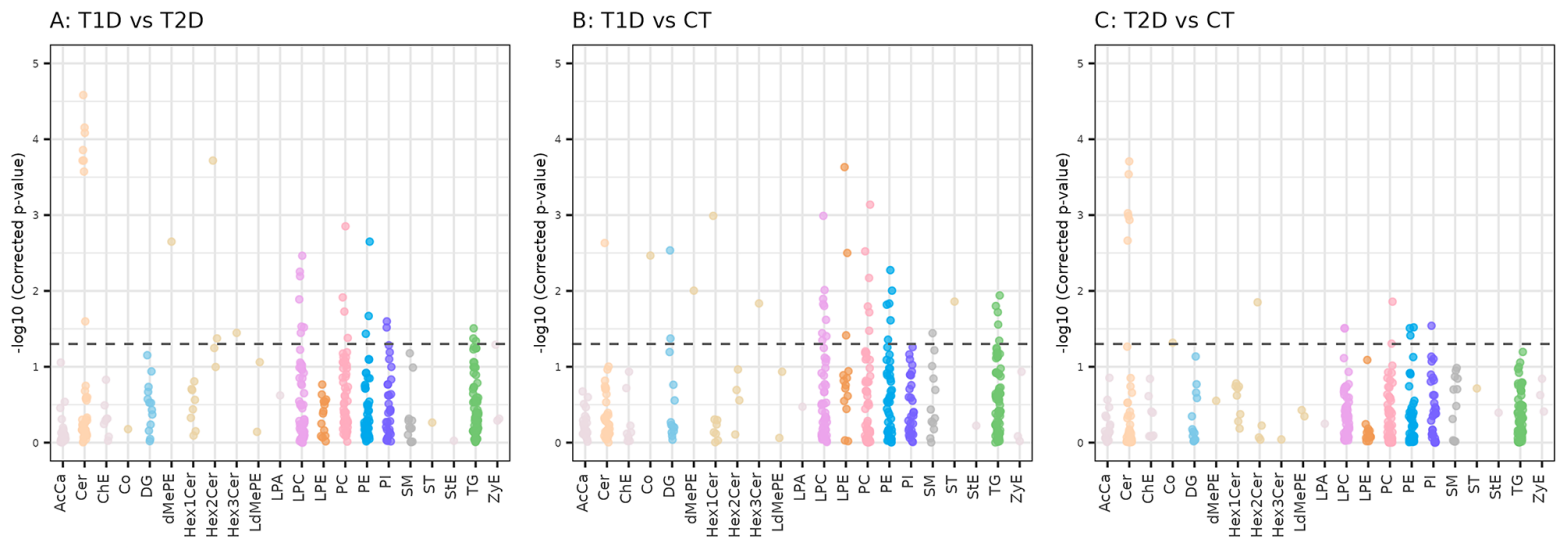
Figure 1 shows the lipid classes that differ in the comparisons according to diabetes status. When comparing both diabetic conditions, lysophosphatidylcholines (LPC) and ceramides (Cer) were more importantly altered than other lipid classes (Fig. 1A). In a similar way, LPCs, phosphatidylcholines (PC), phosphatidylethanolamines (PE) and TGs were especially altered in T1D (Fig. 1B), as well as Cer in T2D (Fig. 1C). Additional File 2 (Table S3) reports the mass-to-charge ratio (mz) and retention time (rt) for each lipid ion significantly associated with one of these conditions in at least one of the analyses. Moreover, the range of corrected p-values, ionization mode and the list of analyses corresponding to the significant corrected p-value is also shown in Additional File 2 (Table S3)

Figure 1. Manhattan plots of the minus logarithm of the corrected p-values (y axis) for each of the lipid classes (x axis) obtained in the analysis of **A**) T1D vs. T2D, **B**) T1D vs. CT and **C**) T2D vs. CT. Corrected p-values are shown for the features that had been annotated using LipidSearch and fulfilled the quality criteria described in the Methods section. The dashed line indicates the threshold of significance ($0.05$). AcCa, acylcarnitine; Cer, ceramide; ChE, cholesterol esther; Co, coenzyme; DG, diacylglycerol, dMePE, dimethylphosphatidylethanolamine; Hex1Cer, hexosylceramides; Hex2Cer, dihexosylceramides; Hex3Cer, trihexosylceramides; LdMePE, lysodimethylphosphatidylethanolamine; LPA, Lysophosphatidic acid; LPC, lysophosphatidylcholine; LPE, lysophosphatidylethanolamine; PC, phosphatidylcholine; PE, phosphatidylethanolamine; PI, phosphatidylinositol; SM, sphingomyelin; ST, sterol; StE, Stigmasteryl ester; TG, triacylglycerol; ZyE, Zymosteryl ester

**Fig. 2:**
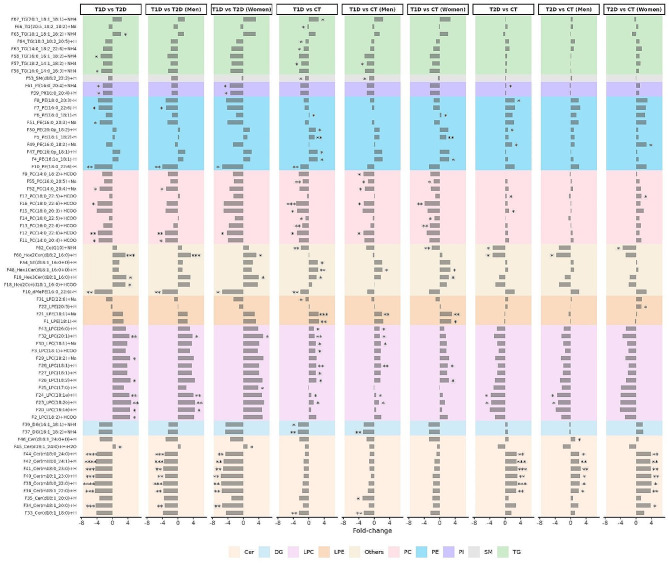
Fold-change values and statistical significance obtained for each lipid determined as being significantly different in at least one of the nine analyses conducted. Statistical significance is indicated using asterisks: corrected p-value (p) < 0.05 (*), *p* < 0.01 (**), *p* < 0.001 (***), *p* < 0.0001 (****). Bars in the right side of each panel indicate a positive fold-change value, while bars in the left indicate a negative one. A positive fold-change indicates that the lipid is increased in the first group (e.g. in T1D vs. T2D, TG(18:1_18:1_18:2) is significantly increased in T1D with respect to T2D). The different colours in the background of the plot show the different lipid classes. In the left, the names of the lipid subspecies are shown. The nomenclatures of the type 16:1e indicate that the fatty acid (FA) of the glycerophospholipid is linked to the glycerol moiety by an ether bond, therefore, the mentioned glycerophospholipid is an ether-glycerophospholipid

In Figure 2, the fold-changes and significance level of the lipids significantly altered in at least one of the analyses are shown.

In general, TGs, sphingomyelins (SMs), PCs, diacylglycerols (DGs) and ceramide lipids were down-regulated in subjects with T1D, while phosphatidylethanolamines (PE) lysophosphatidylethanolamines (LPEs) and LPCs were mainly up-regulated when compared to CT. On the other hand, some LPCs were down-regulated in T2D subjects and ceramide lipids were mostly up-regulated with respect to controls. Results comparing T1D and T2D support the observed opposing effects seen in comparisons against the control group. A generalized opposite disruption of ceramide lipids and LPCs in subjects with T1D and subjects with T2D could be detected. Contrary to LPCs, ceramide lipids were mainly upregulated in T2D with respect to T1D. Specifically, the ceramide lipids increased in T2D were 1-deoxyceramides (i.e., Cer(m18:1_22:0), Cer(m18:0_22:0), Cer(m18:1_23:0), Cer(m18:0_23:0), Cer(m18:0_24:1) and Cer(m18:0_24:0)) (Fig. 2).

**Fig. 3:**
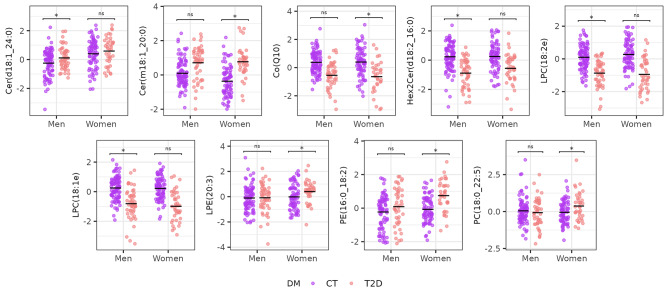
Boxplots of lipids that are significantly associated with T2D in men or women. The nomenclatures of the type 16:1e indicate that the fatty acid (FA) of the glycerophospholipid is linked to the glycerol moiety by an ether bond, therefore, the mentioned glycerophospholipid is an ether-glycerophospholipid

**Fig. 4:**
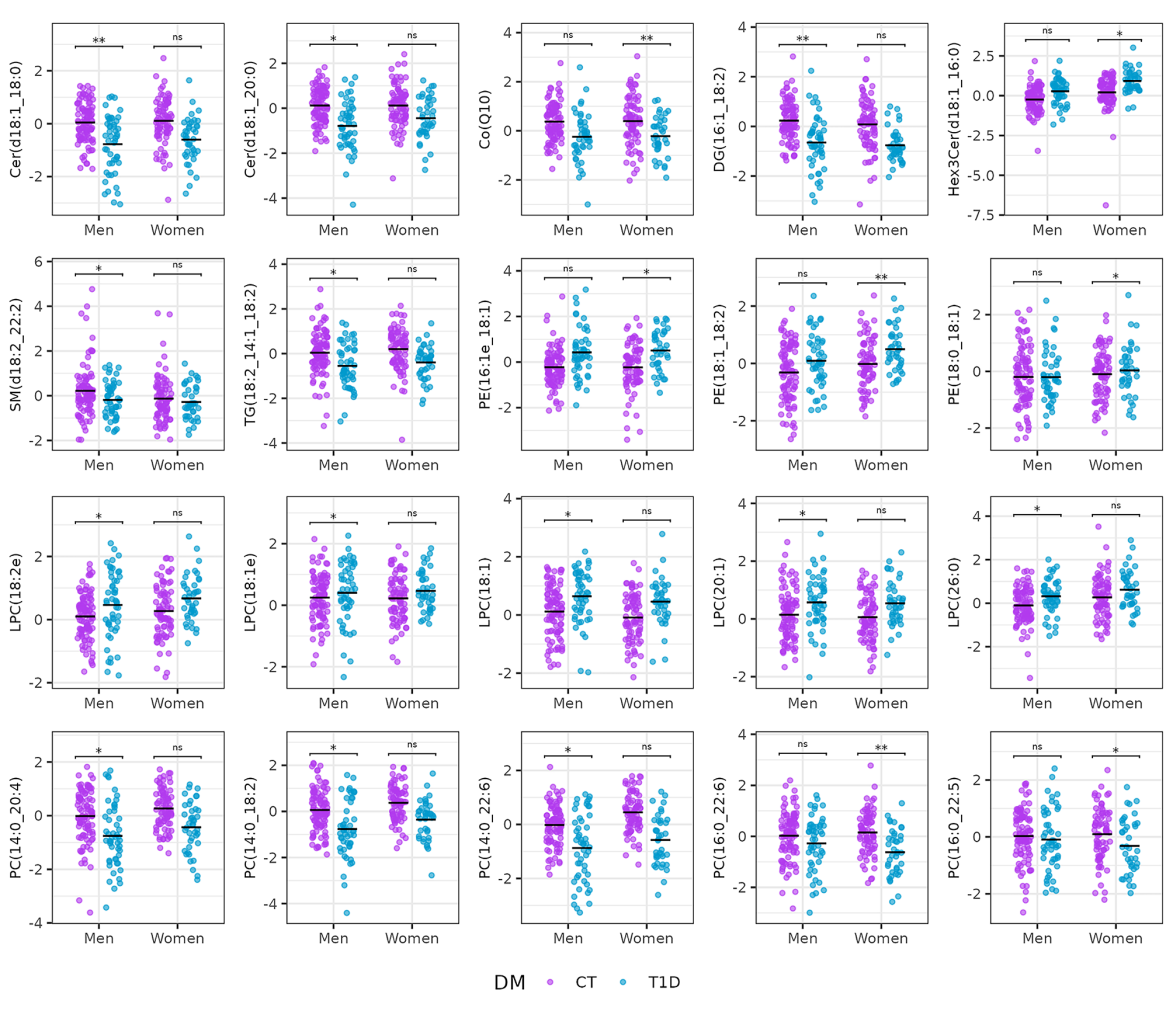
Boxplots of 20 selected lipids significantly associated with T1D in men or women

Several sex-specific lipidomic differences were detected, as shown in Fig. 2. These alterations are further illustrated in Figs. 3 and 4 for T2D and controls comparison and T1D and controls comparison, respectively.

In general, our results showed that PC levels were higher in normoglycemic women than their male counterparts.

**Fig. 5:**
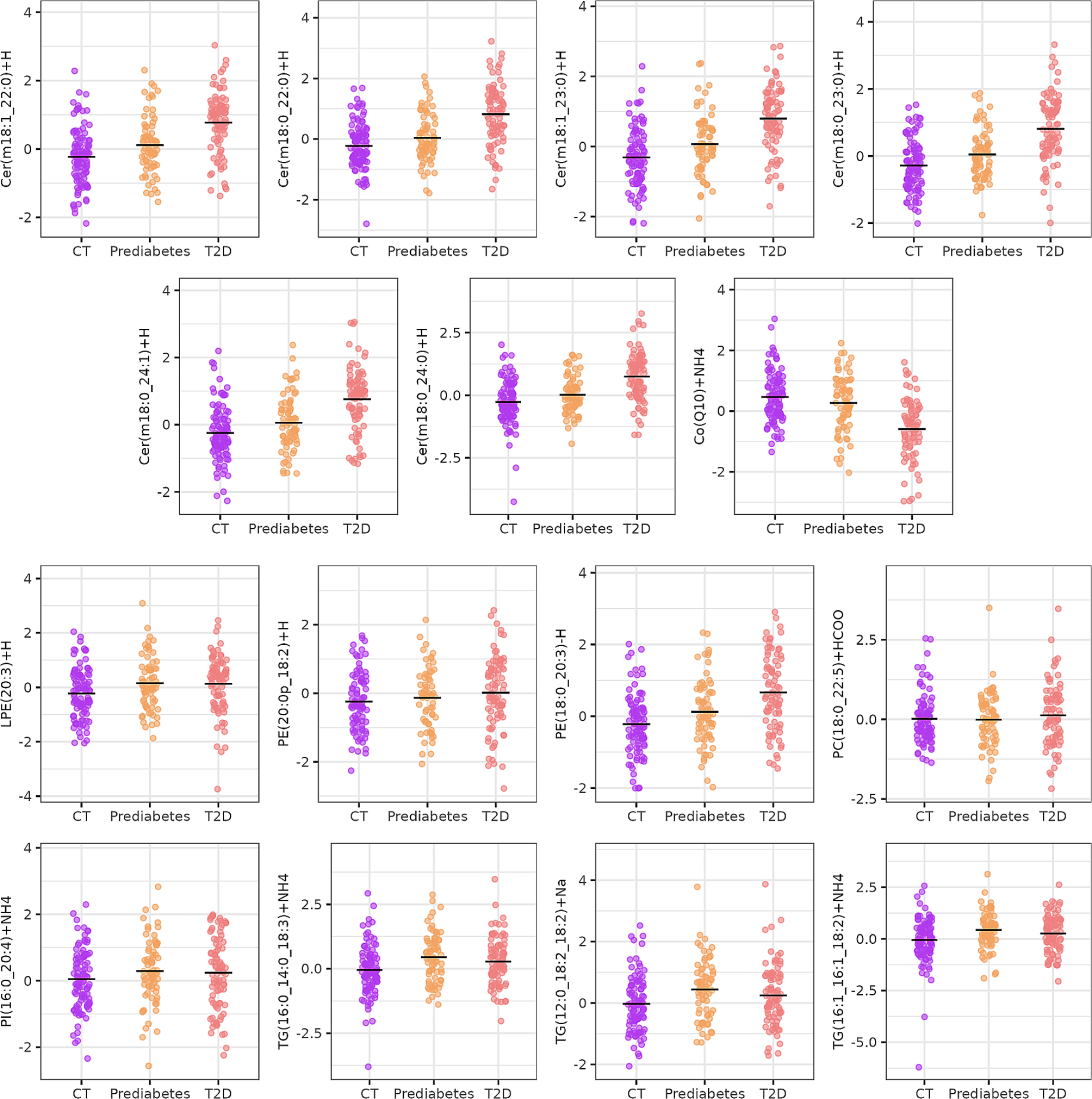
Boxplots of lipids significantly associated with the numeric variable defined as 0 for normoglycemia, 1 for prediabetes and 2 for T2D

Figure 5 shows lipids significantly associated with a numeric variable that describes glycaemic stag, by considering the prediabetes stage. Although Co(Q10) was not statistically significant (q-value = 0.07), the boxplot shown in Fig. 5 shows a gradual decrease through the glycaemic progression.

Our results show a consistent alteration of ceramides, revealing a gradual increase of these lipids in the stage of prediabetes to T2D.

## Discussion

Through an untargeted lipidomic serum profiling approach, we investigated the lipidomic alterations in type 1 and type 2 diabetes; the sex-specific differences in these diseases and the glycaemic state by considering the prediabetes stage. The results reported in the present study have revealed 54 lipid species belonging to 15 different lipid classes potentially implicated in well-known mechanisms involved in type 1 and type 2 diabetes.

### Lysophosphatidylcholine acyltransferase (LCAT) activity

Our results have revealed a panel of LPCs significantly increased in T1D, specifically LPC(26:0), LPC(20:1), LPC(18:1) and LPC(18:2). These findings are in agreement with other studies, where LPC(18:3) has been consistently found to be positively associated with T1D or in those at risk [7]. The mechanisms behind this increase are unclear; however, SMs have been described as inhibitors of LCAT, enzyme responsible of LPCs and cholesteryl esters synthesis from PCs [35]. In our study, SM(d18:2_22:2) was significantly reduced in T1D subjects, which could be a potential explanation for an enhanced activity of LCAT and a subsequent increase of LPC levels. Furthermore, we observed a general decrease of a set of PC species in subjects with T1D. This aligns with some studies that showed a negative association between PC levels and T1D risk [36, 37], and concurs with the suspected enhancement of LCAT activity. The potential consequences of these changes should also be considered. Serum levels of LPC(18:1) and LPC(18:2) were found to be significantly higher in children with T1D after a diabetes ketoacidosis or a hypoglycaemic episode [38], suggesting an association between certain diabetes complications and LPCs.

Although non-significant, our results showed a reduction of LPC levels in T2D compared to controls, and a significant reduction of LPC(20:1) and LPC(18:2) when compared to subjects with T1D. Moreover, PC(18:0_22:5) and PC(18:0_20:3) were significantly increased in subjects with T2D versus controls. Our results are aligned with other studies that showed a positive association between PCs and T2D risk [39], and suggested a reduction of LCAT activity in T2D [40, 41] and a negative correlation between LCAT activity and HbA1c [40–42]. A potential mechanism behind this relationship is the non-enzymatic glycation of apolipoprotein A-I in HDL particles due to hyperglycemia [40, 42].

### Polyunsaturated fatty acids (PUFAs)

Our findings also showed a general down-regulation of TG and DG containing PUFAs in subjects with T1D when compared to controls and subjects with T2D. The decrease of oleic (16:1) and palmitoleic (18:1) acids, as well as a reduction in the synthesis of omega-6 and omega-3 PUFAs in T1D has been previously described. This reduction is caused by decreased activity of Δ6 desaturase and the stearoyl-CoA desaturases (SCD). mRNA transcription of these desaturases is activated by the protein SREBP-1c, the expression and activation of which is modulated by insulin and therefore, reduced in T1D [43]. This mechanism could be an explanation for the significant reduction of PUFA-containing TGs and DGs in subjects with T1D. This becomes even more feasible if we consider that all the TGs and DGs found to be significantly reduced in our study, contain at least one of the previously mentioned fatty acids (16:1, 18:2, 18:3 and 20:5).

### Ether lipids

Peroxisomal defects causing decreased levels of serum ether lipids have been associated with neurodegenerative diseases, cancer, obesity, hypertension [44] and T2D [45]. Plasmalogen PEs containing PUFA have been associated with lower risk of T2D [46]. However, in the present study, PE(20:0p_18:2) was significantly increased in subjects with T1D and T2D when compared to controls, and PE(16:0p_18:1) and PE(16:1e_18:1) were significantly increased in subjects with T1D. On the other hand, previous studies have revealed reduced levels of ether PCs in lean subjects with T2D [45] and in HDL particles of subjects with T2D [9], as well as inverse associations between ether PCs and T2D risk [39]. Moreover, ether PCs have been positively associated with longevity [47]. Although our results do not show a significant association between ether PCs and diabetes, we have found lower levels of ether LPCs (LPC(18:1e) and LPC(18:2e)) in subjects with T2D, supporting the previously hypothesized peroxisomal defect associated with T2D.

### Phosphatidylethanolamines and Lysophosphatidylethanolamines

Regarding phosphatidylethanolamines (PEs), we showed an increase of PE(18:0_20:3) and PE(16:0_18:2) in subjects with T2D. Previous work has reported increased serum levels of PEs in T2D obese subjects. The mechanism proposed to explain this alteration was a higher abundance of PEs in VLDL particles compared to HDL particles, and a relative increase of VLDL particles in obese T2D subjects [45]. Furthermore, another study showed enrichment of PE(38:5), PE(38:6), and PE(40:7) in HDL particles in patients with T2D and CHD [9]. This could explain the increase in PEs observed in the present study, since the lipid species observed in lipidomics mainly stem from circulant lipoproteins. Additionally, PEs have been described as modulators of inflammation and apoptosis [47]. In line with this, our results revealed two PE species, PE(18:0_18:1) and PE(18:1_18:2), significantly increased in subjects with T1D, and interestingly one of their metabolic products LPE(18:1) also increased in T1D. On the other hand, PE(18:0_22:6) was significantly decreased in subjects with T1D, as well as one of its plausible products, LPE(22:6).

### Sphingolipids

The direct inhibition of the insulin-signalling pathway caused by sphingolipids has been widely described. Ceramides accumulation interferes in the insulin-stimulated activation of protein kinase B (Akt/PKB), which decreases glucose uptake in skeletal muscles and activates gluconeogenesis and glycogenolysis in the liver. On the other hand, several studies support the theory that elevated intracellular levels of sphingolipids may hinder mitochondrial respiratory chain activity, thus causing alterations in mitochondrial metabolism [48]. Our results revealed an increase of a set of 1-deoxyceramides, Cer(m18:1_22:0), Cer(m18:0_22:0), Cer(m18:1_23:0), Cer(m18:0_23:0), Cer(m18:0_24:1), Cer(m18:0_24:0), in subjects with T2D compared to subjects with T1D and controls. Moreover, this significant association is maintained when comparing normoglycemia, prediabetes and T2D, showing a gradual increase with the glycaemic state. Related to this, it is quite relevant that we found coenzyme Q10, Co(Q10), to be significantly decreased in subjects with T2D compared to controls. Co(Q10) has a key role in the electron transport chain of the mitochondria and its deficiency in subjects with T2D has been previously described [49]. This concurs with our results and is probably related to the above-mentioned mitochondrial dysfunction. Even though this molecule has not been found significantly associated in the prediabetes analysis (q-value = 0.07), the corrected p-value is close to significance. The plot of its progression has been added to the main manuscript, and it is possible to see a non-linear gradual decrease from normoglycemia to T2D.

### Sex-specific metabolic changes in type 1 and type 2 diabetes

It has been shown that women have a steeper age-related increase of ceramide levels [50]. The loss of oestrogens during and after menopause has been proposed as the main mechanism behind this pattern [50, 51], but other processes have been proposed, such as the differences in sex steroids or the higher levels of oxidative stress in post-menopausal women [51]. Moreover, pre-menopausal women have better cardiovascular health and CVD outcomes than men, but this tendency changes during and after menopause. Ceramides might have a key role in this process, due to the strong relationship between oestrogens and sphingolipid metabolism and the association of ceramides with apoptosis, oxidative stress, inflammation and endothelial dysfunction [52]. Further, menopause has been associated with an increased risk of T2D [53]. In our study, the age of the female subgroup with T2D was 57.3 (SD: 10.6) years. Therefore, we might assume that in a large proportion of the subjects, menopause was playing a role in the lipidomic differences observed. We found one 1-deoxyceramide, Cer(m18:1_20:0), significantly associated with T2D in women but not in men, and in general, the fold-changes and the significance level of the significant 1-deoxyceramides in T2D were higher in women than in men (Fig. 2). Diabetes has been shown to attenuate the protective effect of the female sex in the development of cardiac diseases and nephropathy [54]. The specific lipids that differ between sexes found in the present study could explain the greater impact of T2D complications in post-menopausal women.

Our results also revealed a greater T1D-associated alteration of ceramide metabolism in men, specifically, Cer(d18:1_20:0) and Cer(d18:1_18:0) were significantly decreased only in men. Reduced levels of very long chain ceramide species have been associated with the development of macroalbuminuria [55], while male sex has been reported as a risk factor for the development of macroalbuminuria associated with T1D [56]. Moreover, we revealed a panel of LPCs significantly increased only in men. It has been shown that LPA and LPCs accumulate in the kidney and promote renal inflammation and tubulo-interstitial fibrosis in diabetic rodent models. Six species of LPAs and LPCs were found to be significantly enriched in the urine, but not in plasma, of people with T2D with nephropathy [57]. The mentioned sex-related lipidic differences could help to explain the worse prognosis of T1D-related diabetic nephropathy in men compared to women.

### Strengths and limitations

Our study has several strengths, such as the large number of covariables used in our linear models to minimize confounding, the untargeted approach that allows for a more comprehensive characterisation of the lipidome in people with diabetes, and the consideration of sex-specific lipid differences associated with diabetes. There are also some limitations. First, our findings have not been validated in an independent cohort, and secondly, the observational nature of our study does not allow us to make causal inference. Therefore, further research is required to assess diabetes progression and its complications. Interestingly, our study shows the need to investigate this matter in a sex-specific manner.

## Conclusions

In conclusion, we detected a panel of lipids associated with T1D and T2D, sex-specific differences in lipid metabolism disruption associated with diabetes and lipids associated with the glycaemic state, by considering the prediabetes stage. A large part of the lipids reported in this study have previously been linked to T1D, T2D and/or their complications in the literature, thus confirming their role in diabetes. Regarding sex-specific differences, we reported several lipid species associated with T2D only in women that have been previously related to menopause. This could help explain an unfavourable prognosis of T2D in women of older age compared to their male counterparts. In a similar way, we have shown a set of lipids associated with T1D only in men that have been previously linked to diabetic nephropathy, potentially explaining the worse prognosis of diabetic nephropathy in men. Our findings point to the need of establishing sex-specific strategies in the management and research on diabetes mellitus and its associated comorbidities and suggest the importance of lipidomics in advancing personalized medicine.

### Electronic supplementary material

Below is the link to the electronic supplementary material.

Additional File 1: Figure S1. Flow chart of the participants recruitment. Table S1. Description of the analyses performed. Table S2. Baseline characteristics for each comparison. Figure S2. Upset plot of the positive acquisition mode results. Figure S3. Upset plot of the negative acquisition mode results.

Additional File 2: Table S3. Mass-to-charge ratio (mz) and retention time (rt) for each lipid ion significantly associated with T1D and/or T2D in at least one of the analyses.

## Data Availability

No datasets were generated or analysed during the current study.
