# Supplementary material for: Lipidome characterisation and sex-specific differences in type 1 and type 2 diabetes mellitus
Source: Cardiovasc Diabetol. 2024 Mar 29;23:109. doi: 10.1186/s12933-024-02202-5 (PMC10981308; doi:10.1186/s12933-024-02202-5)

Supporting Information - Lipidome characterisation and sex-specific differences in type 1 and type 2 diabetes mellitus

Figure S1. Flow chart of the participants recruitment.

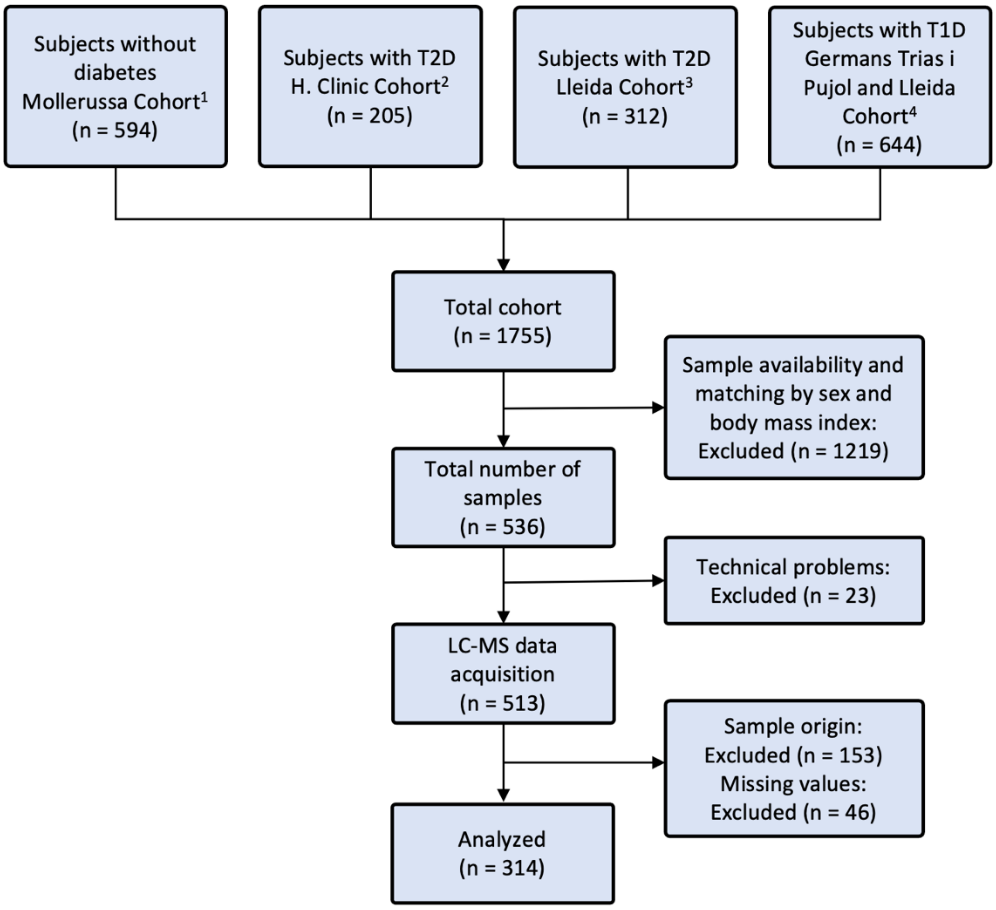

(1) *Vilanova MB, Falguera M, Marsal JR, Rubinat E, Alcubierre N, Castelblanco E, Granado-Casas M, Miró N, Molló À, Mata-Cases M, Franch-Nadal J, Mauricio Di. Prevalence, clinical features and risk assessment of pre-diabetes in Spain: The prospective Mollerussa cohort study. BMJ Open 2017;7.*

(2) *Catalan M, Herreras Z, Pinyol M, Sala-Vila A, Amor AJ, Groot E de, Gilabert R, Ros E, Ortega E. Prevalence by sex of preclinical carotid atherosclerosis in newly diagnosed type 2 diabetes. Nutrition, Metabolism and Cardiovascular Diseases 2015;25:742–748.*

(3) *Alonso N, Traveset A, Rubinat E, Ortega E, Alcubierre N, Sanahuja J, Hernández M, Betriu A, Jurjo C, Fernández E, Mauricio D. Type 2 diabetes-associated carotid plaque burden is increased in patients with retinopathy compared to those without retinopathy. Cardiovasc Diabetol 2015;14:1–9.*

(4) *Carbonell M, Castelblanco E, Valldeperas X, Betriu À, Traveset A, Granado-Casas M, Hernández M, Vázquez F, Martín M, Rubinat E, Lecube A, Franch-Nadal J, Fernández E, Puig-Domingo M, Avogaro A, Alonso N, Mauricio D. Diabetic retinopathy is associated with the presence and burden of subclinical carotid atherosclerosis in type 1 diabetes. Cardiovasc Diabetol 2018;**17**:1–10.*

Table S1. Description of the analyses performed.

Comparisons	Analysis code	Result
T1D vs T2D	R11	P-values associated to T1D and T2D differences
	R12	P-values associated to T1D and T2D differences in women
	R13	P-values associated to T1D and T2D differences in men
T1D vs CT	R21	P-values associated to T1D and CT differences
	R22	P-values associated to T1D and CT differences in women
	R23	P-values associated to T1D and CT differences in men
T2D vs CT	R31	P-values associated to T2D and CT differences
	R32	P-values associated to T2D and CT differences in women
	R33	P-values associated to T2D and CT differences in men

Table S2. Baseline characteristics for each comparison. Data are mean (SD) for continuous variables and number (%) for categorical variables. For continuous variables, the p-values are obtained using a student's t-test and for categorical variables, a chi-squared test. BMI, body mass index; DM, diabetes mellitus; HbA1c, glycated haemoglobin; sBP, systolic blood pressure; dBP, diastolic blood pressure; TG, triglycerides; HDL, high-density lipoprotein; LDL, low-density lipoprotein; aMED, adherence to Mediterranean diet; eGFR, estimated glomerular filtration rate.

	T1D N=59	T2D N=86	P-value	Control N=100	T1D N=59	P-value	Control N=100	T2D N=86	P-value
**Sex (men)**	27 (45.8%)	46 (53.5%)	0.456	60 (60.0%)	27 (45.8%)	0.115	60 (60.0%)	46 (53.5%)	0.456
**Age (years)**	50.7 (9.86)	58.0 (10.0)	<0.001	53.1 (12.3)	50.7 (9.86)	0.164	53.1 (12.3)	58.0 (10.0)	0.003
**Hypertension (yes)**	22 (37.3%)	48 (55.8%)	0.043	17 (17.0%)	22 (37.3%)	0.007	17 (17.0%)	48 (55.8%)	<0.001
**Dyslipidaemia (yes)**	30 (50.8%)	49 (57.0%)	0.577	26 (26.0%)	30 (50.8%)	0.003	26 (26.0%)	49 (57.0%)	<0.001
**BMI (kg/m^2)**	25.5 (3.62)	31.7 (5.62)	<0.001	25.7 (3.62)	25.5 (3.62)	0.797	25.7 (3.62)	31.7 (5.62)	<0.001
**Waist circumference (cm)**	89.1 (12.2)	106 (12.4)	<0.001	94.5 (11.7)	89.1 (12.2)	0.007	94.5 (11.7)	106 (12.4)	<0.001
**DM duration (years)**	24.6 (10.4)	10.6 (7.74)	<0.001	.	24.6 (10.4)	.	.	10.6 (7.74)	.
**HbA1c (%)**	7.58 (0.94)	8.16 (1.54)	0.005	5.26 (0.28)	7.58 (0.94)	<0.001	5.26 (0.28)	8.16 (1.54)	<0.001
**Glucose (mg/dL)**	154 (79.4)	169 (61.2)	0.214	88.0 (7.14)	154 (79.4)	<0.001	88.0 (7.14)	169 (61.2)	<0.001
**Smoking (former/current)**	31 (52.5%)	52 (60.5%)	0.437	41 (41.0%)	31 (52.5%)	0.212	41 (41.0%)	52 (60.5%)	0.012
**sBP (mmHg)**	130 (19.0)	139 (18.2)	0.002	122 (14.6)	130 (19.0)	0.007	122 (14.6)	139 (18.2)	<0.001
**dBP (mmHg)**	71.3 (9.74)	76.9 (10.6)	0.001	76.6 (9.99)	71.3 (9.74)	0.001	76.6 (9.99)	76.9 (10.6)	0.867
**TG (mg/dL)**	68.9 (38.1)	127 (65.8)	<0.001	105 (50.6)	68.9 (38.1)	<0.001	105 (50.6)	127 (65.8)	0.012
**HDL-cholesterol (mg/dL)**	65.5 (16.8)	49.0 (12.7)	<0.001	58.0 (15.2)	65.5 (16.8)	0.005	58.0 (15.2)	49.0 (12.7)	<0.001
**LDL-cholesterol (mg/dL)**	102 (20.2)	103 (33.8)	0.931	128 (29.2)	102 (20.2)	<0.001	128 (29.2)	103 (33.8)	<0.001
**aMED**	4.36 (1.61)	4.19 (1.51)	0.523	3.38 (1.81)	4.36 (1.61)	0.001	3.38 (1.81)	4.19 (1.51)	0.001
**eGFR (mL/min/1.73 m^2)**	91.7 (14.3)	89.8 (19.2)	0.484	87.2 (14.9)	91.7 (14.3)	0.06	87.2 (14.9)	89.8 (19.2)	0.314
**Insulin (yes)**	59 (100%)	31 (36.0%)	<0.001	0 (0.00%)	59 (100%)	<0.001	0 (0.00%)	31 (36.0%)	<0.001
**Metformin (yes)**	0 (0.00%)	68 (79.1%)	<0.001	0 (0%)	0 (0%)		0 (0.00%)	68 (79.1%)	<0.001

Figure S2. Upset plot of the positive acquisition mode results. The codes used for each analysis are shown in Table S1. The bars in the left of the plot indicate the number of significant features found in each analysis. The central matrix shows the different intersections between analyses and the top bars show the number of significant features for each intersection combination, e.g., the first column describes that the analyses R11 and R12 have found the same 205 significant features, while the last column specifies that there is one significant feature found in R21, R31, R32 and R33.

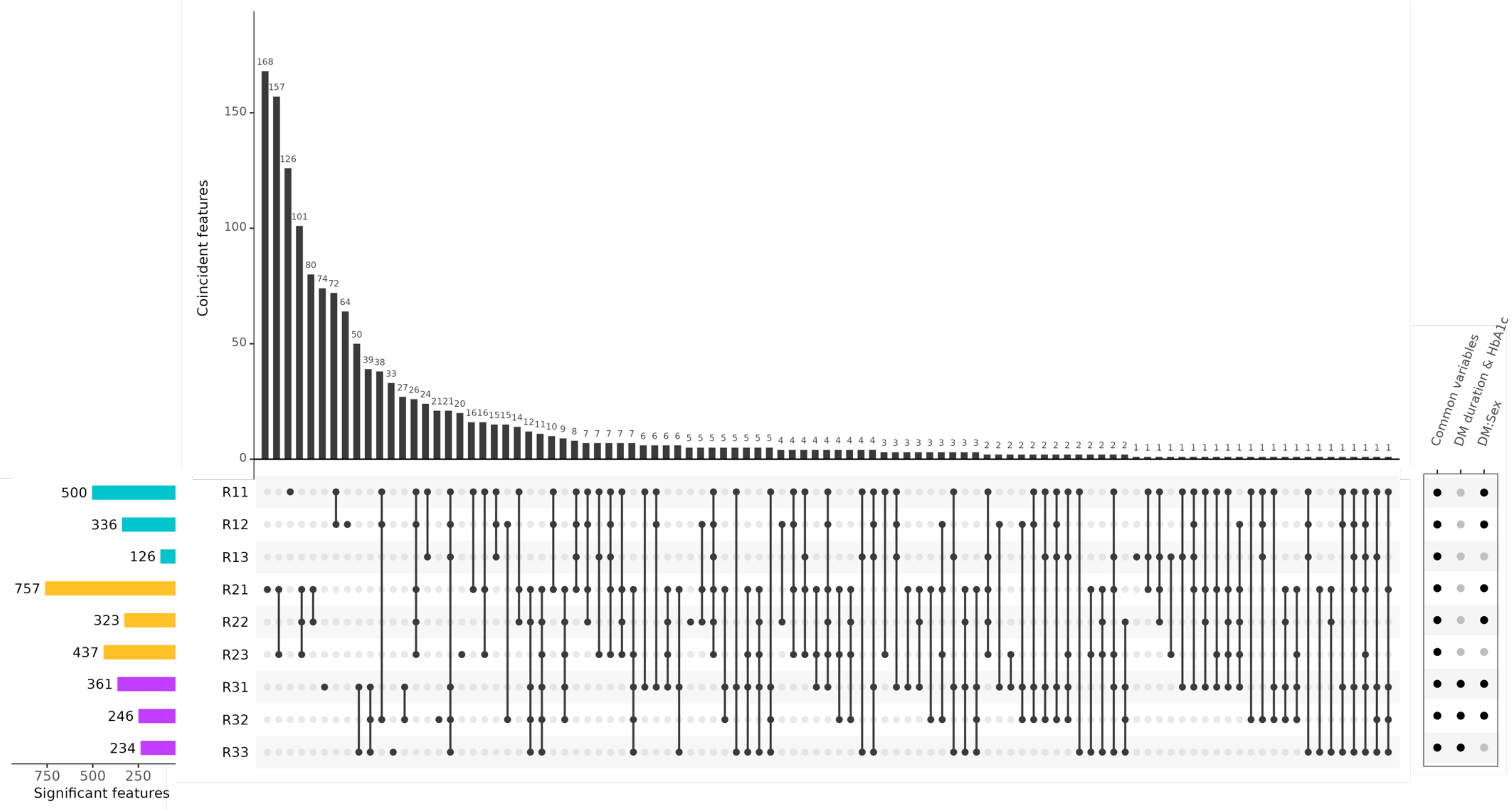

Figure S3. Upset plot of the negative acquisition mode results. The bars in the left of the plot indicate the number of significant features found in each analysis. The central matrix shows the different intersections between analyses and the top bars show the number of significant features for each intersection combination.

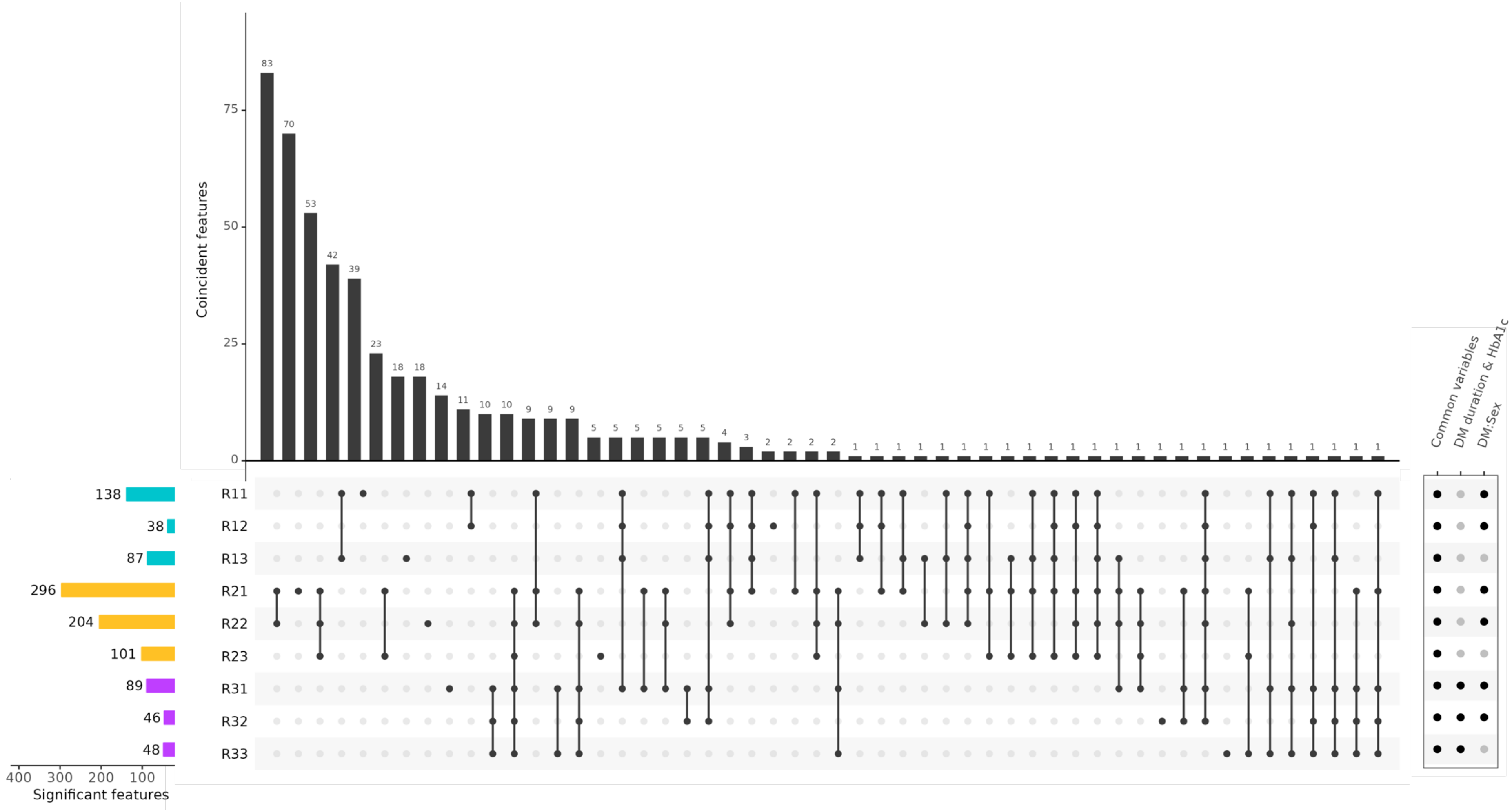

Supplement: Supplementary file 1 — Additional File 1: Figure S1. Flow chart of the participants recruitment. Table S1. Description of the analyses performed. Table S2. Baseline characteristics for each comparison. Figure S2. Upset plot of the positive acquisition mode results. Figure S3. Upset plot of the negative acquisition mode results. [file 12933_2024_2202_MOESM1_ESM.pdf]
